# A focus group study exploring necessary competencies and contextual factors for effective antimicrobial stewardship on dairy farms

**DOI:** 10.3168/jds.2024-25302

**Published:** 2025-03-03

**Authors:** Devon J. Wilson, Elizabeth M. Parker, Rafael Portillo-Gonzalez, Pamela L. Ruegg, Gregory G. Habing

**Affiliations:** 1 Chilliwack, BC, Canada, V4Z 1H3; 2 Department of Veterinary Preventive Medicine, The Ohio State University, Columbus, OH 43210; 3 Department of Large Animal Clinical Sciences, Michigan State University, East Lansing, MI, 48824

**Keywords:** qualitative, education, antimicrobial use, dairy cattle

## Abstract

There is a need for improved antimicrobial stewardship on dairy farms, and changes will be largely enacted by the farm workers who are responsible for carrying out farm procedures. For this reason, efforts directed at educating and enabling farm workers in antimicrobial stewardship are necessary for compliance with farm-level antimicrobial usage policies. Therefore, the objectives of this research were (1) to determine the competencies needed by dairy farm workers to implement antimicrobial stewardship and to inform educational resource development and (2) explore the farm contexts that influence farm worker antimicrobial stewardship capabilities. Initially, focus group discussions were conducted in person with 6 groups of veterinarians at 3 independent conferences held in the United States. To gain additional perspectives, 2 focus groups were conducted via video link with dairy producers from the midwestern United States. The focus groups had 4 to 8 participants and 2 to 4 facilitators each, and were audio recorded and transcribed for analysis. Discussions were audio recorded, transcribed, and evaluated using inductive thematic analysis, with 9 key themes identified. Participants emphasized that farm workers needed to be willing to learn, to be responsible, and to be consistent in their execution of tasks. Furthermore, farm workers needed knowledge about the rationale behind antimicrobial use strategies and disease mitigation practices. General skills needed by farm workers included good communication, excellent observation, and the ability to follow protocols and keep records. Technical skills required by farm workers related to the handling of animals, attention to cleanliness, ability to properly administer pharmaceutical products, and farm equipment maintenance. The skills and knowledge required differed among farm workers depending on their responsibilities and experience, with greater expectations for herd managers compared with employees who specialized in a specific task such as milking. Farm contexts that affected antimicrobial stewardship included the farm size, which had an impact on the 3 other themes: workplace culture, leadership, and tools. Workplace culture encompassed having an approachable environment and clear responsibilities, and leadership encompassed managerial capacity and veterinary support. Important tools for effective antimicrobial stewardship included relevant and up-to-date protocols, technology, equipment, and a proficient workforce. The evidence provided through these focus groups is useful for informing competency-based educational efforts aimed at improving antimicrobial stewardship on dairy farms.

## INTRODUCTION

There is a need for more holistic implementation of antimicrobial stewardship (**AMS**) in animal agriculture to combat emerging antimicrobial resistance (**AMR**), and dairy farms are no exception. Effective AMS initiatives on dairy farms rely on collaboration between producers and veterinarians to implement disease prevention and evidence-based antimicrobial usage (**AMU**) practices, which vary depending on farm size and structure (Ruegg, 2022). Successful initiatives must reach the workers in charge of implementation, which is an increasingly diverse group that often includes nonfamily and immigrant labor (Mills et al., 2021).

A review of 35 studies that aimed to assess the perceptions of veterinarians and dairy farmers regarding AMR and AMU found that improving worker knowledge about AMR and skill development on prudent AMU practices were key recommendations (Farrell et al., 2021). Competency-based education (**CBE**), primarily used in healthcare settings, posits that learning is best measured by the demonstration of mastery (Biemans et al., 2004). Competencies are defined as “connected pieces of knowledge, skills, and attitudes that can be used to adequately solve a problem” (Baartman et al., 2007, p. 5), and competencybased teaching can help ensure healthcare professionals graduate with the “skills and attitudes needed to function properly in a workplace” (van Griethuijsen et al., 2019, p. 517). Given that farm workers often perform animal examinations, administer treatments, and enact disease prevention strategies, CBE could be an effective tool to ensure farm workers are proficient antimicrobial stewards. To design effective CBE tools, there is a need to better document and understand the specific attitudes, knowledge, and skills needed for dairy farm workers.

Recent studies have evaluated AMS interventions that provided education to farm workers through delivery of learning modules (Garzon et al., 2023) or through peer learning groups (Morgans et al., 2021). Although these educational strategies show promise, knowledge alone may not be enough to drive behavioral change (Fishbein, 2008). A review by Garforth (2015) emphasized that farmer behavior is influenced by their experiences and the characteristics of their farms. Social science tools can help understand the barriers and enablers that affect farmers’ ability to adopt AMS practices and inform the development of more effective interventions.

Given the importance of understanding both the necessary AMS competencies for farm workers and the farm contexts that affect their ability to practice responsible AMU, the objectives of this qualitative study were 2-fold: (1) explore veterinary and dairy producer perspectives about the competencies needed to achieve successful AMS on dairy farms, and (2) explore the farm contexts that influence farm workers’ capacity to practice prudent AMS.

## MATERIALS AND METHODS

### Qualitative Approach

This qualitative research was undertaken to collect diverse and nuanced data on competencies relevant to AMS that are tailored to dairy farm workers, and focus groups were chosen to elicit broad views by promoting discussion (Krueger and Casey, 2000). Reporting of the study followed the Consolidated Criteria for Reporting Qualitative Research (Tong et al., 2007). A realist paradigm was adopted, which acknowledges that, as per realism, “there is a real world that exists independently of our perceptions, theories, and constructions” that can be observed by the researcher (Maxwell, 2012, p. 5). Therefore, the perspectives discussed by participants are “real” and could be used for developing education resources.

### Positionality

The research team members were veterinarians and researchers specializing in AMU in animal agriculture, with a particular focus on the dairy industry. Input was received on the research approach and questionnaire design from an expert in curriculum development. The first author (DW) is a female veterinarian with a PhD in veterinary epidemiology and experience conducting focus group studies as a research consultant.

### Reflexivity

The training of the researchers gave good insight into the technical aspects of AMU and knowledge of how to effectively explore diverse perspectives. However, additional insights were needed from veterinarians and farm managers to develop and understand the practical competencies that are applicable to modern dairy farms. Participants were aware of the professional background of the researchers, potentially fostering collegial and open discussion among the veterinary focus groups, but possibly hindering farmers from expressing controversial views. This risk was mitigated because the farmers were involved in other research projects with coauthors (GH, PR, and RP), which likely fostered additional trust. Given the researchers’ background, the findings likely emphasize competencies and contexts that relate to animal health and welfare.

### Participants

Veterinary participants were recruited from 3 conferences for bovine veterinarians in 2023 in the United States (the National Mastitis Council Regional Meeting in Atlanta, GA, the American Association of Bovine Practitioners Recent Graduate Conference in Knoxville, TN, and the American Association of Bovine Practitioners Annual Conference in Milwaukee, WI), where the focus groups were conducted. Veterinarians from a wide geographic range were targeted for the first round of focus groups because of their experience working on multiple farms, which affords a broad perspective on factors that affect AMS. Researchers contacted 115 veterinarians via email after stratifying for 6 different regions in the United States (Northeast, Midwest, Southwest, West, Pacific, and Southeast) or Canada, and selecting randomly among the registered conference attendees using a random number generation function (rand) in Excel (Microsoft, Redmond, WA). Thirty-eight veterinary participants were ultimately recruited, with the 6 focus groups having 7, 6, 4, 6, 8, and 7 participants each. Veterinary participants were not renumerated for their participation.

Following these groups, dairy producers from Ohio and Michigan were purposively recruited based on their proximity to the collaborating universities, their managerial roles, and their ongoing involvement in AMU monitoring trials conducted by the same researchers. Given their familiarity with the research team from these trials, participants were more likely to have considered AMS competencies and perceive research to be beneficial, resulting in more open discussion. Conversely, these farmers likely brought a progressive approach to AMS, which might not reflect the general dairy farming population. Twenty-four dairy producers who had been participating in a companion project focused on evaluating AMU were contacted by email or text message and the 2 focus groups had 7 and 6 producer participants, all of whom were offered a $100 gift card for their involvement.

### Focus Group Discussions

The 8 focus groups for this study took place between January 2023 and February 2024. The first 6 sessions with veterinarians were held in person and the remaining 2 focus groups were conducted with dairy producers via web link (Zoom Video Communications Inc.). The first 4 focus groups were moderated by a consultant with a background in curriculum development, and the remaining 4 groups were moderated by DW. Both moderators were trained in qualitative methods and leading focus groups and used the same discussion guide. The guide was developed collaboratively between GH, PR, and the external consultant, and aimed to explore the communication and problem-solving skills and knowledge needed by dairy farm workers, along with competencies needed for disease diagnosis, prevention, and treatment procedures (Supplemental File S1; see Notes). Besides the primary moderator, 2 to 4 additional research personnel were present observing and helping with the room or technical setup. The average focus group took place over 51 min and ranged from 42 to 64 min. All groups were audio recorded with permission from all participants, and the recordings were transcribed by a professional transcription service and then verified for accuracy against the audio files by DW. Analysis of the transcripts was undertaken using the qualitative software NVivo (version 2022, release 1.6.1; QRS International). The number of focus groups was determined based on data saturation, which was evaluated following Hennink et al. (2019). Specifically, after coding the first 5 focus groups, only 2 new codes were identified in the sixth group, suggesting sufficient data saturation to inform our research questions. Given that dairy farm managers may have a more direct and practical perspective of on-farm needs, additional groups were conducted with this stratum with a goal of deeper understanding of themes. Hennink et al. (2019) suggested that there is little benefit to adding more than 2 additional groups of a different strata, which was consistent with our findings because no new themes were identified during analysis of these groups (Guest et al., 2020).

### Data Analysis

The focus group transcripts were thematically analyzed using the approach by Braun and Clarke (2006) to explore farm contexts that affect worker competencies in AMS. Field notes were neither collected nor used in this analysis, and transcripts and results were not returned to participants for review. Initially, 2 authors (EP and DW) familiarized themselves with the data through repeated reading of the transcripts and collaboratively developing a codebook from the 6 veterinary focus groups using open coding (McDonald et al., 2019). The codebook evolved throughout the coding of the veterinary groups and included a label, descriptive definition, and an exemplary quotation (DeCuir-Gunby et al., 2011). The codebook was applied by DW to the dairy producer focus groups and revised to include additional nuances. Themes were then developed inductively by searching for patterned responses, which was led by DW with input from coauthors until agreement was reached. Validity measures included daily memos (records detailing decision making), peer debriefing with EP, and consultation with other coauthors throughout data analysis and manuscript preparation (Creswell and Miller, 2000).

### Data Presentation

**Figure 1.**
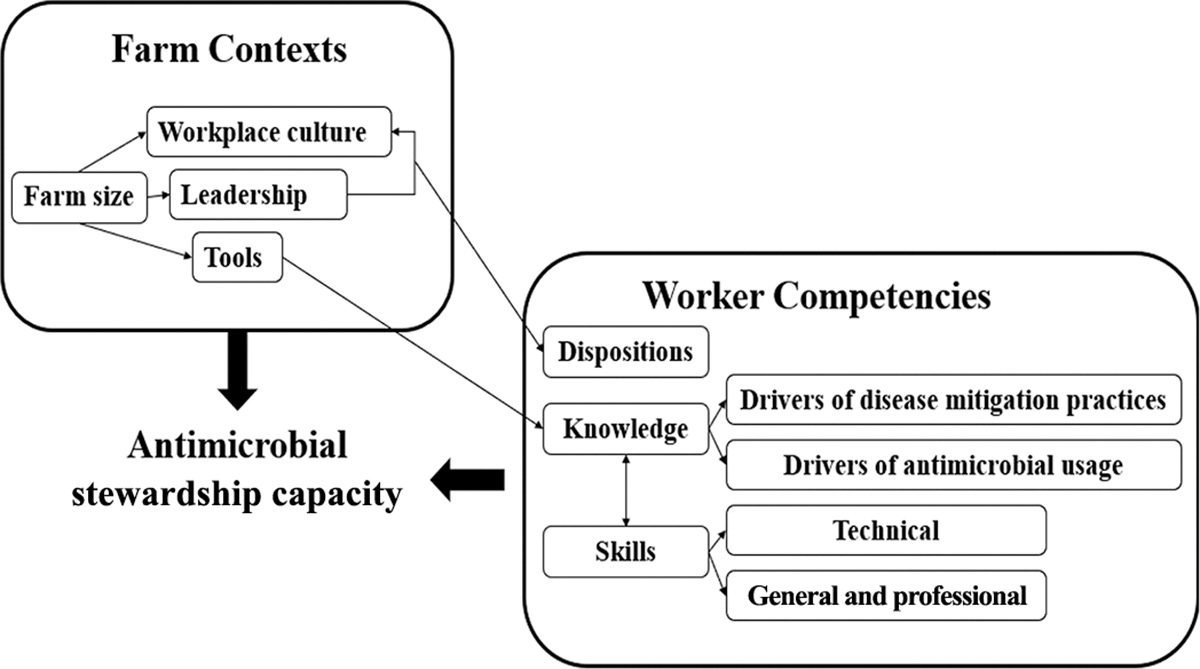
Thematic map of the dispositions, knowledge, and skills required by dairy farm workers to be competent in AMS and the contexts that affect this capacity.

Themes are presented as a map (Figure 1), and using exemplar verbatim quotations, with the speaker identified by focus group (G) and participant number, separated into veterinary (V) and farmer (F) participants (e.g., G1-V1). Clarifications are provided in brackets, and ellipses were used to shorten quotes whenever possible. To maintain a succinct manuscript and because worker competency themes were less nuanced and complex, supporting quotes are presented in Supplemental Table S1 (see Notes).

## RESULTS

### Participants

Focus groups all included male (n = 31) and female (n = 20) participants, with at least 2 of each gender in 6 of the 8 groups. One veterinary focus group contained 1 male and 3 females, and one farmer focus group contained 1 female and 6 males. All participants were involved on dairy farms as a herd or consulting veterinarian in the United States (n = 34) or Canada (n = 4) or held a role on a dairy farm in the midwestern United States that involved management or training of farm workers.

### Thematic Structure

Five key themes encompassed the worker competencies needed, and 4 interrelated themes were identified around farm contexts that affect the AMS capacity. These themes and their relationships are represented in a thematic map (Figure 1).

### Worker Competencies

#### Theme 1—Worker Dispositions.

The dispositions of dairy farm workers were discussed in all focus groups where participants described farm worker “attitude” or “care” as being important to their success in executing prudent AMU, emphasizing 3 subthemes listed below.

##### Subtheme 1.1—Willingness to Learn:

Participants described the importance of farm workers being open to learning and asking questions. Some participants felt that farm workers demonstrated this through being caring and motivated and receiving instruction positively.

##### Subtheme 1.2—Initiative and Personal Responsibility:

Participants described the importance of farm workers being self-starters who are accountable for tasks, especially related to animal care. Some stressed the need for farm workers to feel responsible for producing food ethically and contributing to the farm’s success.

##### Subtheme 1.3—Consistency:

Most focus groups discussed the need for farm workers to be consistent in completing expected tasks. Specific tasks were mentioned, including animal examinations and treatments, recordkeeping, animal handling, and milking procedures.

#### Theme 2—Knowledge of Drivers of Antimicrobial Practices: “The Why.”

Many participants emphasized the need for farm workers to understand the reasoning behind the AMU practices expected at their workplace to encourage consistent compliance with AMS efforts. Four key areas of knowledge are listed as subthemes below.

##### Subtheme 2.1—Milk and Meat Quality and Regulation:

To ensure compliance with meat and milk withholding times for pharmaceuticals, farm workers must understand the risks of drug residues. These risks can render products unsafe for consumption, might trigger a regulatory investigation, and ultimately could prevent milk sales. Workers should also recognize risks associated with off-label drug use, treating large numbers of animals or critically ill ones, and unintentional feed sharing. Knowledge of the farm’s quality assurance program regulations is also essential.

##### Subtheme 2.2—Routes of Disease Transmission Including Zoonoses:

Disease prevention strategies on dairy farms are informed by how pathogens spread, so participants felt that workers should be able to identify common disease transmission routes. Three routes of disease transmission were emphasized, including (1) iatrogenic spread, for example mastitis pathogens from milker’s hands or calf pathogens on clothing, (2) environmental contamination including seemingly clean surfaces because of biofilms, for example, in the maternity pen, on milking equipment, or on calf feeding equipment, and (3) contact among animals including between older and younger cattle, sick and healthy cattle, and with new herd introductions. Farm workers should recognize their personal risk of zoonotic disease exposure (e.g., *Salmonella* spp., *Cryptosporidium parvum*) through touching contaminated surfaces and drinking unpasteurized milk.

##### Subtheme 2.3—Disease Etiology:

Participants generally felt dairy farm workers needed basic knowledge about disease causes and processes, with some expecting workers to differentiate between bacterial versus viral diseases, inflammatory versus infectious clinical signs, and acute versus chronic disease. In some cases, additional knowledge was expected for mastitis, where specialized workers should be capable of identifying pathogens like *Streptococcus uberis*, *Streptococcus dysgalactia*, *Staphylococcus aureus*, or simply grampositive versus gram-negative bacteria, depending on the farm protocols.

##### Subtheme 2.4—Adverse Effects of Antimicrobials:

Background knowledge related to adverse effects of AMU was expected, including gut flora disruptions and AMR.

#### Theme 3—Knowledge of Disease Mitigation Practices: “The What.”

Participants emphasized the need for dairy farm workers to be knowledgeable about methods to mitigate disease, including treatment (subtheme 1) and prevention (subtheme 2).

##### Subtheme 3.1—Rational Veterinary Product Use:

Farm workers were expected to have broad knowledge of treatment practices, particularly distinguishing antimicrobials, vaccines, and anti-inflammatories. Some participants felt minimal pharmacological knowledge was needed, while others expected workers to be able to link treatments with disease types, including infectious, inflammatory, nutritional, and metabolic diseases. Some participants also expected workers to recognize that treatment choices are influenced by disease severity, chronicity, and likelihood of cure. Workers should also be aware that some products, mainly vaccines, are used preventively.

##### Subtheme 3.2—Prevention Strategies:

Following an understanding of disease transmission routes (Subtheme 2.2), participants identified that farm workers needed to know disease prevention methods for cattle including scrubbing boots, hand washing, pasteurizing calf milk, and wearing appropriate protective equipment like clean gloves. Environmental and animal cleanliness was also discussed, with emphasis on procedural knowledge related to teat disinfection, calf bottle disinfection, general farm equipment cleanliness, disinfectant application, water temperature requirements, bedding and ventilation management, and routine testing for diseases of interest to the farm like bovine leukosis or Johne’s disease. Farm workflow was frequently mentioned, and animal movement knowledge was emphasized, including procedures for quarantining new herd introductions, separating sick animals, and disposing of carcasses. Last, several farmers referenced the need for farm workers to know typical cow routines and minimize disruptions for disease prevention.

#### Theme 4—General and Professional Skills.

Professional and general skills farm workers should possess were discussed within 5 subthemes: (1) skilled communication, (2) observation, (3) following protocols, (4) recordkeeping, and (5) the ability to synthesize information to make good decisions.

##### Subtheme 4.1—Communication:

The most frequently discussed professional skill for AMS competency was the ability of farm workers to engage in open and ongoing communication, acting as a team member and asking for help as needed. This includes communicating in a culturally appropriate, respectful, and honest manner; voicing concerns; asking questions; and listening to feedback. Participants relayed the benefits of shared language among personnel, with reading and writing skills and the ability to use farm-specific channels of communication being essential. Farm manager communication was also highlighted, as participants emphasized the need for managers to listen actively and facilitate inclusive team meetings.

##### Subtheme 4.2—Observation:

Participants across focus groups emphasized the need for farm workers to quickly detect abnormalities in animal health and farm operations, including monitoring cattle appearance, behavior, and farm technology data. Farm workers should notice a broad array of visual (Table 1), olfactory (diarrhea, metritis, and ketosis), and tactile (udder texture) signals. It was also noted that farm workers should be especially attentive during periods of high risk for disease, like after calving and in the preweaning period. Farm workers were also expected to recognize equipment abnormalities, especially related to milking and calf feeding.

##### Subtheme 4.3—Follow protocols:

The ability of dairy farm workers to follow approved protocols was seen as paramount for AMS. Participants stressed that farm workers must know or be able to locate and follow protocols for treating, monitoring, culling, or euthanizing animals, and call for help when needed. When treatment is warranted, precise adherence to procedures, including administering and recording the correct drug, dosage, and duration, was deemed essential.

##### Subtheme 4.4—Recordkeeping:

Detailed record-keeping ability was deemed essential by all focus groups. Participants felt that records should be legible and accurate, and include the date, animal identification, disease diagnosis, and the name, dose, and route of the drug administered. Recordkeeping was considered required for regulatory adherence and informative for decision-making.

##### Subtheme 4.5—Synthesizing Data to Guide Decisions:

Participants acknowledged that it was important for farm workers to use information from observations, physical exams, diagnostic tests (e.g., milk culture), weather forecasts, and animal records to make decisions. Other data-based factors to consider included milk and reproductive performance, rumination, and for calves, their drinking speed. Different situations requiring this skill included (1) selecting the right protocol, (2) prioritizing treatments, (3) deciding whether euthanasia or culling is warranted, (4) identifying a need for more information, and (5) recognizing a need to seek help.

#### Theme 5—Technical Skills.

Several technical abilities of dairy farm workers were described for effective AMS, frequently related to disease diagnosis, treatment, and prevention, which fell under 5 subthemes. These technical competencies were role-specific, with higher expectations for farm workers in charge of treating animals compared with those solely employed for milking.

##### Subtheme 5.1—Veterinary Product Administration:

The most common technical skill discussed was the ability to properly store, handle, and administer pharmaceuticals. Farm workers should maintain products, especially vaccines, at the correct temperatures and reconstitute them properly. They must ensure cleanliness of syringes, needles, and administration sites and follow correct dosages and durations. Workers should be proficient in administering products via intramuscular, subcutaneous (including aural), intramammary, and oral routes. They should also identify common products, understand drug labels, and use appropriately sized needles. Last, workers should adhere to withdrawal procedures and maintain records after administration.

##### Subtheme 5.2—Physical Examination:

Farm workers were expected to evaluate clinical signs and disease severity through a thorough physical examination, using sight, smell, palpation (including rectal), and basic tools like a thermometer and stethoscope. Workers should identify signs such as fever, decreased suckle reflex, abnormal feces, painful gait, dull demeanor, abnormal lung or gastrointestinal sounds, abnormal discharge from the nares or reproductive tract, and inflammation in the mammary gland, joints, navel, or eyes. Some participants felt competent workers should diagnose conditions like pneumonia, enteric disease (scours, indigestion, bloat), mastitis, reproductive disease (metritis, retained placenta), dystocia, navel infection, septicemia, milk fever, ketosis, lameness, and knuckled limbs. A few also noted the value of workers using scoring systems to assess calving and calf health, for example, the University of Wisconsin’s Calf Health Scorer and University of California, Davis, Bovine Respiratory Disease system.

##### Subtheme 5.3—Use of Diagnostic Tools:

Most focus groups discussed the need for farm workers to collect samples and use diagnostic tools, such as blood or urine to test for ketones, or milk for culture or antibiotic residue testing. In some cases, farm workers were expected to submit milk samples to a diagnostic laboratory, while others needed to discern between gram-negative, grampositive, or no-growth results, and identify pathogens like *Staphylococcus aureus*. Many participants emphasized the importance of using farm data as a diagnostic tool, including drinking speed, feed intake, ruminations, activity, and metrics related to milk such as conductivity and production.

##### Subtheme 5.4—Cleanliness:

All focus groups discussed the need for workers to have hygienic interactions with animals and maintain a clean environment, including proper teat preparation for milking and intramammary injections, using clean veterinary equipment, and wearing protective equipment such as aprons, boots, or gloves. Participants emphasized clean maternity pens, calf hutches, and calf feeding equipment, including correct application of disinfectants.

##### Subtheme 5.5—Equipment Maintenance:

Participants relayed the importance of farm workers being skilled in farm maintenance procedures to prevent disease. Specific tasks relevant to a worker’s role might include maintenance of milking equipment, soap dispensers, water heaters, robotic feeders, and feed mixers, or simply reporting broken equipment appropriately.

##### Subtheme 5.6—Animal Handling:

A few participants noted that farm workers should be proficient in moving and restraining cattle safely and effectively to reduce stress and prevent disease.

### Farm Contexts

Contextual factors were interrelated, and impacted the competencies needed by dairy farm workers.

#### Theme 6—Farm Size.

Farm size was a key theme, with advantages and disadvantages described for both smaller and larger farms. This context was interrelated with other farm factors, as participants described how larger dairy farms typically use advanced technology and have standard operating procedures. In contrast, G4-V3 explained, “A lot of the (dairy farms) that I’m working at are just pretty small. A lot of them don’t have written protocols for various things.” The lack of technology was not considered inevitably negative, as G5-V3 explained, “Smaller farms, they usually have other ways of recognizing, [for example] that a cow’s off feed, like [in] the tiestall farm where I’m like, ‘hmm, she didn’t clean up her feed.’ Freestalls are going to have a lot more difficulty with that.” Regarding leadership, G3-V6 noted that smaller dairies sometimes lacked veterinary care, saying, “We have some smaller farms. And so particularly I find with those ones where there’s not as much of that vet-client relationship, we’re not out there regularly for herd healths.” Furthermore, G1-V1 described, “The larger dairies, there’s consultants from industry and all over the place that are willing to come in and help them with training employees and helping them develop protocols and stuff.” Related to the working environment, G1-V5 explained, “I think it’s tough around smaller dairies. There’s not enough employees to where you have some specialized employees that would be looking at those sick cows.” Generally, AMS competencies should be tailored to the size of the operation, where workers on smaller farms require keen technical skills and the ability to accomplish a broad set of tasks. In contrast, larger farms may require more general and professional skills for following protocols and using advanced technology, along with technical abilities in role-specific tasks.

#### Theme 7—Tools.

Access to appropriate tools on a dairy farm can help direct appropriate AMU and shape the necessary worker competencies. Relevant tools help optimize farm processes and include basic equipment, up-to-date technology, and current, accessible operational protocols.

##### Subtheme 7.1—Equipment and Technology:

Participants indicated that dairy farms need basic equipment, including diagnostic tools like a California Mastitis Test paddle, thermometer, and stethoscope. On this, G1-V4 said, “It’s the farm’s job to provide those employees in charge of diagnosing with the basic materials they’ll need.” Some participants also felt that farms should adopt the latest technology for recordkeeping and monitoring cattle health and performance via metrics like cow activity, rumination, and milk production. As G3-V2 said, “I think that the farm owner and/or managers should be up-to-date on the latest technology, and try to keep their farms . . . whether it’s innovative or what.” Effective use of these tools requires farm workers to be properly trained, ensuring competent AMU.

##### Subtheme 7.2—Relevant Protocols:

Relevant and accessible protocols were discussed in all focus groups, including the need for reviewing protocols regularly with a veterinarian and key employees to “figure out what the best protocols are” (G7-F4). Having written protocols available in Spanish and English, and using visual aids was considered valuable, and directly relates to subtheme 4.3 (ability to follow protocols). For example, G1-V5 specified, “I think the other thing that’s really helpful on dairies of all sizes too is to have pictures of their particular facility in whatever they’re doing.” Establishing protocols was seen as key to prudent AMU, although participants stressed that effective implementation was more important than the protocols themselves. As G5-V1 stated, “That word protocol covers a wide range of different things, some of which are pieces of paper that are meaningless and some of which are things that really help people to do their job.”

#### Theme 8—Leadership.

Modern dairy farms depend on trained and proficient personnel, making the leadership of managers and veterinarians an important determinant in a farms’ AMS. The leadership resources available to dairy farms were influenced by farm size and helped shape the farm culture (Figure 1).

##### Subtheme 8.1—Managerial Capacity:

Participants relayed the need to have capable leaders on the farm directing AMS efforts, which could include farm owners or managers, veterinarians, and external consultants. First, participants felt leaders on dairy farms needed to be capable of training workers to diagnose and treat diseases appropriately, often taking personal ownership for the task, as G7-F6 explained, “I feel that boils down to me as a manager . . . because if something happens, it’s my fault ultimately for not giving the proper training to them.” Many participants felt it was imperative to prioritize teaching “why” tasks should be completed, and to use case-based, experiential learning. As G8-F2 said, “I have found in my experience that employees are more likely to follow through with what they’re supposed to do if they know why they’re doing it.” Training was seen as most useful for ensuring prudent AMU when it was ongoing, especially considering staff turnover, as G3-V3 said, “There is, I think, some significant lacking of training . . . which then leads to delayed treatments because they don’t know how to identify things. And then probably leads to overtreatment because that calf then has just chronic illness.” Effective leaders on dairy farms were also described as being successful in delegating tasks and monitoring outcomes. For example, G1-V6 said, “Ultimately the manager is responsible for all the treatments, but he’s not going to administer every single one. . . . He has to delegate certain techniques or chores to other workers . . . but also follow through and make sure all that stuff is being done correctly.” Participants also felt leaders should listen to worker feedback and critically evaluate farm processes when considering areas for investment, as G3-V2 explained, farm owners or managers should be “having that continual internal questioning of like, ‘are we doing this the best we can?’ I think [this] is huge for farms to truly move forward and become better.” Overall, having farm leaders capable of training, delegating, and overseeing antimicrobial treatments was a key context for workers to become competent antimicrobial stewards.

##### Subtheme 8.2—Veterinary Support:

Veterinary participants felt they had an important role in training, monitoring, and helping workers develop skills and knowledge and make good antimicrobial treatment choices, as G4-V1 said, “While the palpations get me on the farm, while I’m there, my job is to help them do their job and to follow up on any animal that’s been on therapy.” In the dairy producer groups, veterinarians were seen as a valuable resource, with G7-F4 saying, “I also would add that working with the veterinarian I think is important. And having a veterinarian that has good communication is also helpful.”

#### Theme 9—Workplace Culture.

Workplace culture was described as a key context in AMS success on dairy farms and was linked to the need for strong leadership. Having an approachable culture that promotes teamwork with clearly defined roles and responsibilities was highlighted as beneficial.

##### Subtheme 9.1—Approachable Environment:

Participants used various ways to describe the importance of having an approachable environment for all personnel. Participant G6-V5 described the need for an environment where “[workers] have comfort in, you know, [their] ability to speak freely. There’s much to be said about the culture in that workplace and how it correlates with milk quality.” Some participants described the need for workers to feel comfortable asking questions, which could be challenging due to a lack of trust, personal pride, or inability to communicate in the same language. Creating a positive environment hinged on good communication and investing time in getting to know people, as F2-V1 said, “I just think it’s really, really huge that managers can communicate with them, or as veterinarians, that we can communicate with [workers] . . . talk to them in a way that’s nonoffensive, that they understand, treat them with deference and respect.” Participant G7-F5 recognized the practical benefits of having a respectful farm culture for improving farm processes, saying, “Listening to [worker] feedback and making sure that they feel comfortable to provide that feedback and provide their thoughts on how something could be improved.”

As a cornerstone for developing a good workplace environment, building rapport between workers and managers was emphasized. As G2-V4 described, “We’ve worked hard at building a relationship with [employees] that helps them know that I care about them, but also that they’re on the same side as me in terms of accomplishing the goal on our farm, but it did take a lot of effort to build that relationship.” This was sometimes described as a need to get to know workers personally, including their cultural identity and traditions. Others described the importance of showing appreciation and helping workers feel as if they are working toward a common goal. In one focus group, G5-V4 described showing appreciation through either verbal encouragement or incentives to ensure workers feel “like part of the team and that they’re important to the company.” Having a good workplace culture was considered an effective way to mitigate poor employee performance and retention and promote AMS efforts.

##### Subtheme 9.2—Clear Responsibilities:

Participants noted that dairy farm workers on small and large farms should have clarity around their responsibilities and roles. Additionally, different proficiency around animal disease mitigation was expected for workers depending on their roles. For instance, F4-V1 said, “For somebody in the parlor, I think just understanding that they’re going to be my first line of defense for clinical mastitis and understanding what that looks like and how to identify that in the cows I think are my big expectations for those employees. And that’s maybe a little bit easier than my fresh pen employees or my hospital employees. I think those guys have a lot more diseases to be able to conquer.” Understanding and respecting these different roles was considered important to prevent conflict arising between employees working in different areas of the farm.

In situations that are beyond their roles, participants felt workers needed clarity around when and how to seek help from a veterinarian or manager. For example, regarding disease treatment, G2-V4 explained, “I think it’s also up to management to give them very straightforward criteria. . . . Define it clearly for them, and then if it falls outside of that, they know to ask for help.” Many participants also described the importance of having achievable expectations that are communicated consistently across managers. As G7-F2 described regarding diagnosing and treating dairy cows, “I don’t think you can just expect somebody to learn that in a short period of time. You either need to have worked alongside somebody for probably years or gone to some kind of college or technical school to learn some of that to really be proficient.” Participant F2-V4 used the example of the speed of cows entering a rotary parlor to demonstrate that success depends on having reasonable expectations, saying “You’re never going to get compliance of protocols if you set them up to fail.”

## DISCUSSION

In this study, we explored competencies needed by dairy farmers to achieve appropriate AMS and the impactful farm contexts. Worker competencies fell under 5 themes: (1) personal dispositions, (2) knowledge of drivers of antimicrobial practices, (3) knowledge of disease mitigation practices, (4) general and professional skills, and (5) technical skills. The competencies differed depending on both the farm worker’s role and the farm contexts, which were divided into 4 main themes: (6) farm size, (7) tools, (8) leadership, and (9) workplace culture, including having an approachable environment and clearly defined roles. Construction of a comprehensive set of educational tools surrounding AMS for farm workers first necessitates a complete listing of the necessary competencies. Subsequently, the adequacy of current educational tools can be assessed and new materials created to fill the gaps. This is the first manuscript that has worked toward this competency set specifically for dairy farm workers. Furthermore, farm leadership will find these results informative for evaluating their implementation of disease prevention and treatment practices.

Because competencies include knowledge, skills, and attitudes required to solve problems (Baartman et al., 2007), the workers’ willingness to learn, consistency, and responsibility were seen as important for accomplishing AMS. This was also reflected in a study on the implementation of AMS strategies in a human healthcare setting, where one pharmacist stated, “You can’t run a stewardship program unless you have people who are very, very, very trustworthy running it” (Pakyz et al., 2014, p. S261). The link between worker dispositions and farm contexts is also clear from the results of our study, because strong leadership and good veterinary care were crucial for enabling day-to-day AMS competency in farm workers. In part, this likely reflects a bias of the participants recruited for this study, who understandably felt their roles were important. To our knowledge, this is the first study to describe how farm managers contribute to success in AMS, including through being capable of training workers, delegating tasks, and monitoring antimicrobial treatments. Efforts for training AMS on dairy farms have been undertaken with successful outcomes (e.g., Garzon et al., 2023), but similar attention has not been devoted to training human resource managers. In an evaluation of employee management on large dairy farms, authors identified several management shortfalls, including inadequate provision of training and feedback and poor communication of goals (Durst et al., 2018). Human resource training for dairy farm managers that improves relationships among the worker hierarchies might help empower the more desirable worker dispositions, including a higher willingness to learn, consistency, and responsibility. Overall, our study highlights the need for a combination of effective leadership and motivated workers to achieve successful AMS on dairy farms.

Building on the importance of leadership and veterinary guidance for AMS success, the role of workplace culture in supporting effective AMS also emerged as a key factor. Research understanding the effect of workplace culture on AMS on dairy farms is limited. However, variability in workplace culture has been documented by Durst et al. (2018), who described differences among farms in employee ratings on teamwork and concluded that communication was a central weakness across farms. Other work has linked poor work environments on dairy farms to animal welfare, noting the influence of factors such as hours, wages, and management (Anneberg and Sandøe, 2019). Considering these studies together, efforts for improving workplace culture could positively affect the execution of AMS on dairy farms.

Knowledge was considered foundational for dairy farm workers to be engaged and committed to AMS. An investigation of Dutch dairy, veal, and pig farmers found that increased knowledge of infection routes and the effectiveness of antimicrobials was correlated with reduced usage of antimicrobials (Kramer et al., 2017). The link between knowledge and disease prevention behavior has also been demonstrated; for instance, a systematic review of infection control procedures in human healthcare showed a strong association between knowledge and compliance (Alhumaid et al., 2021). Knowledge around specific disease mitigation strategies was a key theme, including knowledge about efficacy of and indications for administration of different pharmaceuticals. Other evidence also suggests a need for knowledge about rational veterinary product use, because approximately one-third of farmers in Scotland believed that viral infections could be treated with antibiotics (Borelli et al., 2023). Regarding disease prevention, Ritter et al. (2017) found that farmers need sufficient knowledge about disease transmission to make effective decisions about prevention strategies.

Participants additionally described several general and professional skills needed for AMS. Communication and observation skills were emphasized, along with the ability to synthesize observations and data to guide decisions. To our knowledge, the link between communication and AMS on dairy farms has not been previously documented. However, a focus group study investigating the implementation of AMS strategies in a human healthcare setting also noted the importance of communication among team members (Pakyz et al., 2014). In their qualitative review, Farrell et al. (2021, p. 4589) recommended technical skill development to “prevent and manage mastitis and other diseases while reducing AMU via improved biosecurity and herd-management measures.” Importantly, the farm worker competencies intertwined with the identified external contexts. For instance, participants in this study acknowledged that the execution of the worker skills was dependent on the availability of the tools, including basic diagnostic tools and more advanced animal monitoring technology. The availability of these tools is further modified by farm size. A recent review by Neculai-Valeanu et al. (2024) described the opportunity to integrate precision data from individual animals to tailor intervention strategies, as conceptualized in precision livestock farming, although the authors acknowledged that the technology costs may limit implementation on small farms. Regardless, technologies that improve animal monitoring remain a promising area for improving AMS (Costa et al., 2021). Participants also felt having and following protocols was important for improving AMS. This perspective aligns with other research that has documented the success of using treatment protocols to reduce AMU without compromising animal health (e.g., implementing algorithm-based treatment for neonatal calf diarrhea, Gomez et al., 2017). In contrast, a qualitative evaluation of barriers for reducing AMU on farms found that even when protocols are available, they may be too general or broad and difficult to use in practice (Cobo-Angel et al., 2021). Participants in this study echoed the need for useful protocols and moving beyond being “pieces of paper.”

Farm leadership is essential for developing worker knowledge and skills, which is also affected by herd size. Farm size was in turn frequently discussed by participants as a determinant of resource availability and workplace culture. Participants described ways in which small and large farms had advantages for AMS, which have not been previously described. However, a review by Robbins et al. (2016) suggested there is no clear relationship between farm size and welfare, but acknowledged that having a larger employee base allows more specialization of individual workers. Studies have found herd size to be positively correlated with AMU in specific instances, such as for injectable cephalosporins in Canada (Saini et al., 2012) and in conventional (but not organic) herds in Denmark (Krogh et al., 2020). The findings of our study underscore the importance of tailoring AMS strategies depending on farm size. For example, advisers who work with larger farms might suggest better use of technology and focus on training specialized workers. In contrast, advisers working with smaller farms may focus on building the capacity of farm owners to individually assess and treat animals systematically. Furthermore, given that smaller farms may lack external expertise and veterinary care, they may especially benefit from educational resources and facilitation of protocol development on AMS. Further evaluation of how veterinarians and other farm leaders can support AMS implementation across different farm types is needed to identify effective strategies for improvement.

The dispositions, knowledge, and skills outlined in this study as necessary for competent AMS were wide-ranging, making it unlikely for entry-level workers and those with broad roles on the farm to excel in all areas. Continuous training is crucial for developing these competencies over time (Garzon et al., 2023). However, achieving this can be challenging, especially in large farms experiencing high employee turnover, as evidenced by Durst et al. (2018), who observed turnover rates ranging from 8% to 144% across 12 study farms in the United States.

Our study only evaluated the perspectives of veterinarians and dairy farm managers from Canada and the United States and cannot be generalized to all contexts. Because our data focused more heavily on the knowledge and skills needed for AMS, further study might elucidate other farm worker dispositions that are associated with good AMS practices on dairy farms. Furthermore, these perspectives do not reflect the farm workers who often implement AMS actions. Farm worker perspectives on the resources needed to implement AMS practices successfully would provide additional insight and be worthy of further investigation. Last, although data saturation was assessed, we considered this analysis to provide meaningful results to inform our research question. Additional insights could have been gained from conducting more focus groups, especially with dairy farmers from a broader geographic area. As with other qualitative work, the lived experiences of the researchers and participants are reflected in the results and should be considered when applying the findings more broadly.

## CONCLUSIONS

Participants in this study identified 5 key competency areas required for dairy farm workers related to AMS and 4 themes related to impactful farm contexts. Farm workers need knowledge of drivers for AMU practices along with knowledge of the procedures used on the farm for disease mitigation. General skills needed by dairy farm workers included communication, observation, and the ability to synthesize data to make decisions, and technical skills included safe and effective handling and examination of animals and administration of treatments. Farm contexts that influence AMS vary across the dairy industry, with differences in farm size, available tools and human resources, and workplace culture. The competencies are important for constructing learning outcomes for educational efforts directed at improving AMS on dairy farms, and the relevant contexts need to be optimized to ensure that skilled, knowledgeable workers can implement prudent AMU practices.

## Supplementary Material

Focus group script

Compentencies evidence

## Figures and Tables

**Table 1.** Visual cues indicating an abnormality that dairy farm workers should be competent in detecting

Animal appearance	Animal behavior	Animal output	Equipment and data

Eye recession	Feed or milk intake	Milk consistency	Milk filter appearance
Eye inflammation	Stance and gait	Milk volume	Ruminations
Ear position	Respiratory effort	Fecal consistency	Drinking speed
Body condition	Ability to rise		Milk production
Gut fill or distension	Attitude change (“slow”)		Cow activity

## Nonstandard abbreviations used:

AMR: antimicrobial resistance
AMS: antimicrobial stewardship
AMU: antimicrobial use
CBE: competency-based education
