# Supplementary material for: A focus group study exploring necessary competencies and contextual factors for effective antimicrobial stewardship on dairy farms
Source: J Dairy Sci. Author manuscript; Available in PMC 2026 Jan 21. (PMC12820904; doi:10.3168/jds.2024-25302)
Supplement: Focus group script [file NIHMS2129875-supplement-Focus_group_script.pdf]

OHIO STATE CURRICULAR FOCUS GROUP SCRIPT

INTRODUCTION AND EXPLANATION

A. Consultant explains purpose of focus group ***(2 minutes)***

As part of our collaborative effort to develop competencies for individuals responsible for the day-to-day care and direct management of lactating cows and/or preweaned calves, we are inviting you to participate in a needs assessment focus group. As part of this focus group, you will be asked to reflect on the competencies necessary for antibiotic stewardship on dairy farms.

I will start by providing you with some information about the focus group. In front of you, you have an informed consent form. As described, the purpose of this focus group is to learn about your experience with antibiotic stewardship and to understand how this information is relevant in your career. We hope to use this information to provide inform the development of the competencies.

B. Explain Focus Group ***(10 minutes)***

We are interested in your opinions. Everything you say in this focus group will be kept private and no names will be used in any reports we produce. It is important to us that you give us your honest opinions, so everything you say will remain confidential. Aside from your time, there are no costs for taking part in this focus group and although we have tried to avoid risks, you may not want to answer some of the questions that are asked of you. You do not have to answer questions you do not want to, so just tell me if you want to skip a question.

The focus group will be short and will only last about one hour. Please speak clearly and share your opinions, so we can capture everything you say. There are no right or wrong answers. Before we begin, I will ask you if you consent to this focus group.

Please take a few minutes to review the focus group consent form. If you consent to participation, please sign the consent form. Also, please indicate whether you consent to audio recording. If you choose not to participate in the focus group, it will not impact your relationship with Ohio State University.

Do you have any questions?

-Pause to answer questions-

IF PARTICIPANTS CONSENT TO AUDIO RECORDING:

As a reminder, the recording will be deleted as soon as the focus group is transcribed. If you do not wish to be recorded that is okay, we can still conduct the focus group and will take notes as we talk. **May I turn on the recorder? (YES OR NO)**

IF YES:

-Turn on recorder-

Okay, great. I have started the recorder.

IF NO: -Do not turn on recorder-

Okay, no problem. The notetaker will take notes as we talk to record our discussion.

Do you consent to this focus group interview? (YES OR NO)

We will begin our focus group discussion now.

C. Focus Group ***(45 minutes)***

Question Number	Question	Notes
1	Describe your professional experience with dairy farm workers responsible for the day-to-day care and management of lactating cows/preweaned calves.	
2	What do you think are the most important communication skills needed by these dairy farm workers?<br><br>*Probe: Tell us about some common situations on the dairy farm that present communication challenges for workers.*	
3	What do you think are the most important problem solving skills needed by dairy farm workers?<br><br>*Probe: Tell us about some common situations on the dairy farm that present problem solving challenges for workers.*	
4	What are your expectations for a dairy farm worker regarding basic pharmacology and drug label knowledge?<br><br>*Probe: How is that knowledge most commonly used on the dairy farm?*	

5	What are your expectations for a dairy farm worker regarding basic microbiology knowledge?<br><br>*Probe: How is that knowledge most commonly used on the dairy farm?*	
6	What are your expectations for a dairy farm worker regarding lactating cow disease diagnosis?<br><br>*Probe: What specific procedures/skills are necessary to be considered competent in this skill?*	
7	What are your expectations for a dairy farm worker regarding preweaned calf disease diagnosis?<br><br>*Probe: What specific procedures/skills are necessary to be considered competent in this skill?*	
8	What are your expectations for a dairy farm worker regarding disease prevention in lactating cows AND preweaned calves?<br><br>*Probe: What specific procedures/skills are necessary to be considered competent in this skill?*	
9	What are your expectations for a dairy farm worker regarding regulations, record keeping, and protocols?<br><br>*Probe: What specific procedures/skills are necessary to be considered competent in these skills?*	

D. Closing ***(3 minutes)***

Is there additional information you would like to share about your experience?

Thank you very much for your participation. Do you have any questions about our focus group?

If you think of anything, please contact us using the phone number provided on the consent form. Once again, thank you for talking to us!
