# Supplementary material for: A focus group study exploring necessary competencies and contextual factors for effective antimicrobial stewardship on dairy farms
Source: J Dairy Sci. Author manuscript; Available in PMC 2026 Jan 21. (PMC12820904; doi:10.3168/jds.2024-25302)
Supplement: Compentencies evidence [file NIHMS2129875-supplement-Compentencies_evidence.pdf]

Table S1: Supporting evidence for competencies identified using veterinary and farmer focus groups for farm worker competencies required for prudent antimicrobial use on dairy farm

**Theme**<br>Subtheme	Quote	Participant
**Worker Dispositions**<br>Willingness to learn	*"being a teachable human… It's not always knowledge. Many times it's attitude and care."*	G4-V1
Initiative and personal responsibility	*"if they observe it, who do they tell? Do they recognize a line of responsibility or do they have to take the responsibility themselves? So, initiative probably."*	G6-V4
	"at the end of the day, they just have to remember that we're producing food."	G7-F2
Consistency	*"able to reliably do their job in a consistent and adequate manner.*"	G5-V1
**Knowledge of drivers of antimicrobial practices**		
Milk and meat quality and regulation	*"if they understand withdrawal side of it, like, there is huge issues if we put that (cow's milk) in the tank before she can go."*	G6-V6
Routes of disease transmission including zoonoses	*"Knowing potential sources. So the reason why we don't want cows calving in their pen is because now a calf is born in poop, rather than in a clean bedded pack. So it's understanding the why behind what they do."*	G2-V4
	"*wash their hands before they (eat). Try not to pick up crypto or something.*"	G4-V2
Disease etiology	*"they ought to know that if it's enteric disease, maybe it's related to something they ate, and if it's respiratory disease, maybe it's related to something they breathed, and if it's either one, it's probably related to immunity"*	G5-V1
	"(on the) mastitis side, what's the difference between … a coliform or, you know, non-coliform, or mycoplasma versus all the other things for mastitis. Those would be ideal for them to know, especially if they're working on the parlor".	G6-V4
Adverse effects of antimicrobials	"*It's not that there's no negatives to giving an antibiotic. If you accidentally give it to somebody that didn't need it, now their gut flora is all messed up*".	G6-V6
	"(farm workers) think, 'Okay, well, this (antimicrobial treatment) will fix the problem.' Well, it'll fix the problem for now, but if you keep doing it and doing it and doing it's not going to fix the problem. It's going to cause resistance."	G4-V2
**Knowledge of disease mitigation practices**		
Rational veterinary product use	"*just basic understanding of classes of pharmaceuticals and what, you know, what is an antibiotic? What is an NSAID*?"	G6-V4
		G7-F3

	"*the reason we have a protocol is because this has proven to work, and extra is not better or less is not worse or anything.*"	G1-V4
	"*an antibiotic is used to treat an infection. So if it's a cow with ketosis or milk fever, why they don't need an antibiotic.*"	
	"*we have workers and owners that (say) 'Why do we vaccinate? Animal's not sick.'*"	G6-V7
	"*Not a lot* (of knowledge about pharmacology). *I mean, mostly just if the animal fits this disease definition, give this drug.*"	G3-V2
	"*if she has been treated for three days but her milk's still abnormal, it's not necessarily because there's bacteria there, it's because there's still inflammation in that gland.*"	G1-V1
	"*like, calf diarrhea, I've certainly had a lot of conversations with farms about we're not just gonna throw antibiotics at them. Maybe if they need it, we will, but most of the time, it's a fluid issue.*"	G6-V5
	"mycoplasma aren't gonna respond to certain antibiotics."	G5-V6
Prevention strategies	"*basic disease prevention, scrubbing boots… Just general farm flow and biosecurity.*" "*Caring for sick calves at the end. You're going to feed everyone and then take care of sick calves, not the other way around. That way you're not spreading disease*."	G3-V5<br>G4-V3
	"*I would say letting the cows be cows, like make sure feed is pushed up so they can eat, make sure beds are able for them to lay down, and make sure they're not standing in a holding ramp for too long. Um, pretty much try not to interrupt their daily life as much as possible*."	G7-F3
**General and professional skills**		
Communication	"*Well, I think the ability to work as a team, and if they're struggling with something to reach out to their fellow coworkers or management when it's appropriate*."	G2-V6
	"(describing a farm that routinely reviews procedures with employees before milking) *that kind of communication skill is really important, especially on big dairies where there's multiple people either handling or treating."*	G1-V5
	"*I think personally the person that's at least administering or managing the people administering should be able to write and read."* G2-V4 added that as a manager they, "*work very closely with (the employees) and I do speak Spanish, which helps the communication aspect greatly."*	G1-V5
Observation	"*prompt identification of sick animals is really critical to ensuring appropriate antibiotic use*."	G1-V1
	"*at least once a week for ketosis for your fresh cows… and during those high risk periods, say pre-weaning or, in that immunity slump after birth kind of thing*."	G3-V4
Follow protocols	"*definitely follow protocols and make sure the protocols are completed, the cow gets treated, and then … the proper thing is entered in… It's very important to follow (protocols) if you're going to be successful*."	G8-F6

Record keeping	"*comfort and competence with (a) wand and an RFID reader and a tablet… Or if it's even just a paper records and a book. They're clear, they're detailed, dates, cow ID, what they were treated with.*"	G3-V5
	"*if you haven't entered the information in properly, um, you can't make decisions going forward*"	G8-F6
Synthesizing data to guide decisions.	"*able to look at all of the information with that animal, its prior treatment history, age or stage of lactation, and then make an assessment as to whether or not it's appropriate to actually treat that animal or not.*"	G1-V1
	"take care of the things that are really important, such as dehydration,"	G7-F4
**Technical skills**		
Veterinary product administration	"*a farm worker must understand the difference between routes. So IM versus SubQ.*"	G1-V6
Physical examination	"*The reason you're doing the whole exam is getting a temperature, checking for ketones, looking at her eyes and be listening to her movement because (an antibiotic will not fix) diarrhea from a DA (displaced abomasum)"*.	G4-V1
Use diagnostic tools	"(when diagnosing mastitis) *I'm not asking them to identify down to the species of bacteria, just make it very easy for them because there's a lot of opportunity for error if you set your expectations too high there."*	G1-V1
Cleanliness	"*We really stress … cleanliness too: how they wipe and fix, how they prep.*"	G2-V1
Equipment maintenance	"*Reporting broken equipment. Reporting the flush is leaking or just basic... If you see something that doesn't seem right, just say something."*	G2-V4
	regarding a situation when a farm worker was washing equipment without detergent, the *"infection rate (went) through the roof."*	G2-V1
Animal handling	"*are they able to safely move a cow to an area to treat her? Are they, like, chasing after her trying to inject something, and it's mostly going in the alley*?"	G6-V6
	"*the key prevention, you know, just keeping a nice, calm, comfortable cow overall.*"	G7-F7
